# Burden of serious fungal infections in the Netherlands

**DOI:** 10.1111/myc.13089

**Published:** 2020-04-28

**Authors:** Jochem B. Buil, Eelco F. J. Meijer, David W. Denning, Paul E. Verweij, Jacques F. Meis

**Affiliations:** ^1^ Department of Medical Microbiology Radboud University Medical Center Nijmegen The Netherlands; ^2^ Center of Expertise in Mycology Radboudumc/CWZ Nijmegen The Netherlands; ^3^ Department of Medical Microbiology and Infectious Diseases Canisius Wilhelmina Hospital (CWZ) Nijmegen The Netherlands; ^4^ The National Aspergillosis Center, Education and Research Centre Wythenshawe Hospital Manchester University NHS Foundation Trust Manchester UK; ^5^ Division of Infection, Immunity and Respiratory Medicine Faculty of Biology, Medicine and Health The University of Manchester Manchester UK; ^6^ Global Action Fund for Fungal Infections Geneva Switzerland; ^7^ Manchester Fungal Infection Group Core Technology Facility The University of Manchester Manchester UK; ^8^ Center for Infectious Diseases Research Diagnostics and Laboratory Surveillance National Institute for Public Health and the Environment (RIVM) Bilthoven The Netherlands; ^9^ Bioprocess Engineering and Biotechnology Graduate Program Federal University of Paraná Curitiba Brazil

**Keywords:** aspergillosis, candidemia, cryptococcosis, epidemiology, mucormycosis, pneumocystis

## Abstract

**Background:**

Fungal diseases have an ever‐increasing global disease burden, although regional estimates for specific fungal diseases are often unavailable or dispersed.

**Objectives:**

Here, we report the current annual burden of life‐threatening and debilitating fungal diseases in the Netherlands.

**Methods:**

The most recent available epidemiological data, reported incidence and prevalence of fungal diseases were used for calculations.

**Results:**

Overall, we estimate that the annual burden of serious invasive fungal infections in the Netherlands totals 3 185 patients, including extrapulmonary or disseminated cryptococcosis (n = 9), pneumocystis pneumonia (n = 740), invasive aspergillosis (n = 1 283), chronic pulmonary aspergillosis (n = 257), invasive *Candida* infections (n = 684), mucormycosis (n = 15) and *Fusarium* keratitis (n = 8). Adding the prevalence of recurrent vulvo‐vaginal candidiasis (n = 220 043), allergic bronchopulmonary aspergillosis (n = 13 568) and severe asthma with fungal sensitisation (n = 17 695), the total debilitating burden of fungal disease in the Netherlands is 254 491 patients yearly, approximately 1.5% of the country's population.

**Conclusion:**

We estimated the annual burden of serious fungal infections in the Netherlands at 1.5% of the population based on previously reported modelling of fungal rates for specific populations at risk. With emerging new risk groups and increasing reports on antifungal resistance, surveillance programmes are warranted to obtain more accurate estimates of fungal disease epidemiology and associated morbidity and mortality.

## 1 INTRODUCTION

Fungal diseases have a significant impact on human health and affect over 1 billion people worldwide.^1^ Disease burden ranges in severity from mild skin, nail and hair infections affecting a high proportion of the global population, to severe invasive fungal infections affecting a smaller immunocompromised population. Current national estimates of more severe invasive fungal infections are lacking but experts believe that the global rate of invasive fungal infections is increasing due to an increasing population receiving immunosuppressive therapy, a growing elderly population and increased survival from previously lethal diseases. Epidemiological knowledge on fungal infections in the Netherlands is limited due to tenuous hospital and national surveillance data. In this study, we estimate the burden of serious (life‐threatening) fungal infections in the Netherlands, in concordance with the globally initiated LIFE programme (www.LIFE-worldwide.org).

## 2 MATERIALS AND METHODS

The annual burden of fungal infections in the Netherlands was assessed using fungal infections frequencies for chronic pulmonary aspergillosis (CPA), allergic bronchopulmonary aspergillosis (ABPA), severe asthma with fungal sensitisation (SAFS) and invasive aspergillosis, cryptococcosis, *Pneumocystis* pneumonia, mucormycosis, *Fusarium* keratitis, candidemia, *Candida* peritonitis and vulvo‐vaginal candidiasis. A literature search was conducted in order to identify specific fungal infection incidences and epidemiological reports from the Netherlands. When epidemiological data was unavailable, the incidence or prevalence of fungal infections was estimated based on the specific population at risk and the reported incidences for these risk groups. For all estimates, the most recent epidemiological data available was obtained for this study. The authors confirm that the ethical policies of the journal, as noted on the journal's author guidelines page, have been adhered to. No ethical approval was required as this is a review article with no original research data.

Population data for 2017 were obtained from Statistics Netherlands (CBS).^2^ The total population in the Netherlands in 2017 was 17 081 507. The female population between 16 and 50 years totalled 3 667 386. The current total patients diagnosed with HIV in the Netherlands (n = 20 800), as well as the proportion of confirmed cases on anti‐retroviral treatment (93%, n = 19 289), and the annual new AIDS cases (n = 724) were retrieved from the HIV monitoring report 2018.^3^ The annual incidence of patients with tuberculosis (n = 484; with and without HIV coinfection) was obtained from a surveillance report on tuberculosis in the Netherlands 2016.^4^ The prevalence of COPD in 2018 (n = 613 800, all stages of disease), as well as the number of hospital admissions in 2017 (n = 33 735) and the total number of patients with asthma in 2018 (n = 636 200), was obtained from the ‘public health and healthcare’ website.^5^, ^6^ The number of children with asthma was estimated to be approximately 100 000. The number of patients with cystic fibrosis in 2017 (n = 905) was retrieved from the ECFS Patient Registry Annual Data Report.^7^ The yearly number of solid organ transplants (n = 1292, of which 998 were renal) was obtained from the ‘Dutch transplant foundation’ website,^8^ averaging data obtained from years 2015‐2017. Exact numbers of related allogenic stem cell transplantations were not available. In 2015, 407 unrelated HSCTs were performed; it is estimated that 1/3 of allogenic HSCTs were from related donors and twice as many autologous HSCTs were performed. Therefore, we used a yearly number of 611 allogeneic and 1,222 autologous HSCTs in the model. The yearly number of patients with acute myeloid leukaemia (AML) (n = 732) was retrieved from the national cancer registry,^9^ where the average of 3 years (2015‐2017) was used to estimate the average number of patients. Total number of intensive care unit (ICU) admissions for 2017 (n = 80 687) was obtained from the ‘National Intensive Care Evaluation’ (NICE) foundation.^10^

The rate of invasive aspergillosis was estimated to be 10% for AML patients, 0.5% in renal transplant recipients, 4% for lung and liver transplants and 6% for heart transplants, based on an epidemiological study from France.^11^ The percentage of invasive aspergillosis in non‐AML patients with haematological disease was estimated to be similar to that for AML patients, including autologous transplantation.^11^, ^12^ The rate of invasive aspergillosis in patients with influenza admitted to the ICU was estimated based on the number of influenza associated pulmonary aspergillosis (IAPA) cases reported for the 2015‐2016 season in Dutch University Medical Centres. A total of 144 patients with influenza were admitted to the ICU and 16% of these patients matched the definition of IAPA.^13^ The ICU beds in the beforementioned Dutch University Medical Centres cover 22% of the total ICU beds in the Netherlands.^14^ The incidence of IAPA in ICU beds in University Medical Centres was extrapolated to the total number of ICU beds in the Netherlands. We estimated that approximately 50% (45/83 or 54%)^15^ of patients with IAPA were immunocompetent and therefore to prevent counting duplicates, only these non‐immunocompromised patients were considered additional cases of invasive aspergillosis. To calculate the rate of azole resistance in invasive aspergillosis, we used the mean resistance percentage (11.3%) based on a national surveillance programme of five University Medical Centres (14.7%) and five teaching hospitals (7.8%).^16^

The incidence of mucormycosis was based on an estimated population incidence of 0.09 per 100 000 population per year (averaged over 10 years).^17^, ^18^ The incidence of *Fusarium* keratitis was calculated using the mean incidence of 0.045 per 100 000 population per year between 2005 and 2016 reported in a recent retrospective study on *Fusarium* keratitis in the Netherlands.^19^ The annual and 5‐year prevalence of CPA in patients with tuberculosis was based on a method described previously.^20^ Tuberculosis as the underlying cause of CPA was predicted to be 25%. The number of ABPA cases in asthma was estimated to be 2.5%,^21^ and the burden of ABPA cases in cystic fibrosis (CF) was estimated to be 18%.^22^, ^23^ The burden of SAFS was estimated to be 33% of the 10% most severe asthmatic adult patients.^24^ The number of invasive aspergillosis cases in COPD patients was estimated based on a previously reported frequency of invasive aspergillosis in admitted COPD‐patients of 1.3%.^25^

The annual incidence of candidemia for the Netherlands is calculated to be 2.61 per 100,000 population. In Europe^26^ and globally,^27^ 5.52 and 6.87 episodes per 1000 ICU admissions are diagnosed with *Candida* bloodstream infections, respectively. *Candida* peritonitis (intra‐abdominal candidiasis) was set to the European average of 1.84 episodes per 1000 ICU patients.^26^ We assumed that 6% of women aged 16‐50 had recurrent vulvo‐vaginal candidiasis.^28^, ^29^, ^30^ The number of HIV patients presenting with *Pneumocystis jirovecii* pneumonia (PJP) and disseminated or extrapulmonary cryptococcosis was estimated by taking the average of reported cases from 2011‐2017.^3^ Non‐HIV related cases of extrapulmonary cryptococcosis were based on numbers from the Netherlands Reference Laboratory for Bacterial Meningitis (AMC/RIVM).^31^, ^32^ Cases of non‐HIV related PJP were calculated using local annual PJP incidences in our university (n = 15) and teaching hospital (n = 9), extrapolated to recently published total number of available patient beds in the Netherlands for academic (n = 6987) and non‐academic (n = 30 766) hospitals.^14^ All medical records of patients with *Pneumocystis jirovecii* positive PCR in bronchoalveolar lavage fluid were retrospectively evaluated from 2017‐2018 for the clinical and microbiological diagnosis.^34^ HIV positive patients diagnosed with PJP were excluded from analysis to prevent duplicates.

## 3 RESULTS

We estimated that the annual burden of serious invasive fungal infections in the Netherlands was 3185 while the total number of debilitating fungal infections was 254 491 (Table 1). Extrapulmonary or disseminated cryptococcosis averaged 9 patients each year, mostly in non‐HIV patients. The average annual proportion of PJP patients co‐diagnosed with HIV totalled 48 patients, while the estimated non‐HIV patient cohort was 692. The total number of patients with invasive aspergillosis was 439 in patients with respiratory disease, 790 in immunocompromised/cancer patients and 55 in immunocompetent patients with severe influenza. Altogether approximately 1283 patients suffered from invasive aspergillosis. Of these cases, we estimated that a total of 145 patients suffered from azole‐resistant invasive aspergillosis. The number of patients with CPA was calculated to be 257.

**Table 1:** Annual burden in the Netherlands of serious fungal infections and other fungal infections significantly affecting quality of life

	Incidence rate/ prevalence proportions per 100 K	Total burden		HIV/AIDS	Respiratory	Cancer/Immunocompromised	ICU
Cryptococcosis, disseminated or extrapulmonary	0.05	9	Incidence	2		7	N/A
Pneumocystis pneumonia	4.3	740	Incidence	48		692	N/A
Invasive aspergillosis	7.7	1283	Incidence		439	790	55^a^
Azole resistant aspergillosis	0.8	145		N/A	N/A	N/A	N/A
Chronic pulmonary aspergillosis	1.5	257	Prevalence		257		
Candidemia	2.6	445	Incidence			N/A	445
Candida peritonitis	0.9	239	Incidence			N/A	239
Mucormycosis	0.1	15	Incidence			15	
Fusarium keratitis	0.045	8	Incidence				
Total serious fungal infection burden	**18.6**	**3185**					
Recurrent vulvo‐vaginal candidiasis	1288	220 043	Prevalence	N/A	N/A	N/A	N/A
Allergic syndromes							
Allergic bronchopulmonary aspergillosis (ABPA)^b^	80.9	13 568	Prevalence		13 568		
Severe asthma with fungal sensitisation (SAFS)^b^	105.5	17 695	Prevalence		17 695		
Total fungal burden	**1444**	**246 675**					

^a^ The number of influenza associated aspergillosis cases was 109 of which 50% already included in immunocompromised column.

^b^ The total number of patients with ABPA and SAFS was reduced with 25% for the calculation of the total fungal burden to prevent duplicates.

Total serious fungal infection burden and Total fungal burden are the sum of the above.

Invasive *Candida* infections had an estimated incidence of 445 and 239 for candidemia and candida peritonitis, respectively. Mucormycosis had an average annual reported incidence of 15. *Fusarium* keratitis had an estimated annual incidence of 8. Recurrent vulvo‐vaginal candidiasis had an estimated prevalence of 220 043. The rate of ABPA in adults with severe asthma and CF was estimated to be 13 568 while the burden of SAFS was 17 695. We reduced the total number of patients with ABPA and SAFS for calculation of the fungal burden by 25% (7816 patients) as some patients with ABPA may also have SAFS.^35^ Altogether, the annual debilitating burden of fungal diseases in the Netherlands was approximately 1.5% of the country's population.

## 4 DISCUSSION

We estimated the annual burden of serious fungal infections in the Netherlands based on previously reported modelling of fungal rates for specific populations at risk. We estimated that the number of patients with invasive aspergillosis, candidiasis, pneumocystis pneumonia, cryptococcosis, candida peritonitis, *Fusarium* keratitis and mucormycosis is 3185. Furthermore, the burden of less severe syndromes, albeit with significant impact on the quality of life like ABPA, SAFS and candida vaginitis, is 251 306. As direct epidemiological surveys reporting fungal infection rates in Dutch patients are not available for most fungal diseases, and infection rates in risk groups may change over time and differ among countries, only rough estimates of disease burden could be calculated. We included the most prevalent serious fungal infections and three fungal diseases with high impact on the quality of life. Estimates of several potential serious infections like phaeohyphomycosis were not described, as no reliable incidence estimates were available and incidences are presumed to be very low. Globally, fungal skin diseases are the 4th most prevalent disease in a global estimation of disease burden in 2010 with close to 1 billion affected people.^1^ Unfortunately, current fungal skin disease estimates for the Netherlands are unavailable. Although we believe to provide an accurate representation of the current burden of debilitating fungal diseases, one has to bear in mind that the total number of fungal infections in the Netherlands is probably significantly higher than the number presented here as cutaneous infections like dermatophytosis and superficial *Candida* infections were not included.

Based on our model, the most frequent underlying conditions of invasive aspergillosis in immunocompromised patients in the Netherlands are cancer, hematopoietic stem cell and solid organ transplantations. However, due to the high prevalence of COPD, respiratory diseases remain an important underlying risk for invasive aspergillosis. In addition to these classic risk factors for invasive aspergillosis, novel conditions associated with invasive aspergillosis have recently been identified. A significant proportion of ICU admitted patients with influenza suffer from IAPA.^13^, ^15^ We estimated the percentage of patients with influenza with aspergillosis to be 16% based on a Dutch retrospective study.^13^ This rate is consistent with a study reporting a rate of 19% of cases with IAPA in 2009‐2016 in seven ICUs in Belgium and the Netherlands.^15^ However, this may be an overestimation as only University Medical Centres participated in this study. The rate of IAPA may vary between years reflecting the severity of the influenza season.^13^, ^15^ In addition, other diseases may temporarily increase the incidence of invasive aspergillosis. There are indications that critically ill patients with SARS‐CoV‐2 may be at risk to develop invasive aspergillosis. Li and Xia described CT findings in patients with SARS‐CoV‐2 suggestive of invasive aspergillosis including halo or reversed‐halo sign.^36^ However, as data on invasive aspergillosis in COVID‐19 patients is currently sparse, it remains uncertain whether the risk of invasive aspergillosis in critically ill patients with SARS‐CoV‐2 exceeds the general risk of invasive aspergillosis in critically ill patients. In total, we estimated the rate of invasive aspergillosis to be 7.7/100 000, which is somewhat higher to that reported by surrounding countries. Belgium estimated the rate to be 6.08/100 000, while the rate in Germany and Denmark was 5.1/100 000.^30^, ^37^, ^38^ Largely, we used the same methodology for calculating the invasive aspergillosis infection rate. However, we have added the number of patients with IAPA while Belgium, Germany and Denmark did not. Regardless, the number of patients with IAPA only accounted for a small proportion of the higher rate (0.33/100 000). The higher estimated rate can be explained by our relatively high number of transplantation and COPD patients. Some risk groups for invasive aspergillosis like lung cancer, which has an incidence of up to 2.6%, were not taken into account.^39^ In addition, we used a frequency of invasive aspergillosis in admitted COPD‐patients of 1.3% which was based on study from Spain.^25^ Another study from China reported a frequency of invasive aspergillosis of 3.9% in admitted COPD‐patients.^40^ The actual number of patients with invasive aspergillosis may be surpassed due to an ever increasing number of solid and hematopoietic stem cell transplantations that are treated with novel treatments strategies with a shorter time to neutrophil recovery.

Here, to the best of our knowledge, we have made the first estimate on the rate of azole resistant invasive aspergillosis. The azole resistance rate in invasive aspergillosis was based on a national surveillance programme incorporating data from five University Medical Centres and five teaching hospitals in 2018.^16^ We used the mean resistance frequency from these hospitals to extrapolate the azole resistance frequency in the Netherlands. We calculated that 145 patients (11.3%) with invasive aspergillosis may be infected with an azole resistant isolate. This is a significant finding as patients with voriconazole resistant invasive aspergillosis had a 20%‐25% increased day‐42 mortality compared to patients with voriconazole susceptible infection.^41^, ^42^ However, the resistance frequency in patients is based on positive cultures, irrespective of presence of invasive aspergillosis. Patients with invasive aspergillosis are often culture‐negative and exact resistance frequencies in patients with invasive aspergillosis are not available. It is unknown whether the resistance frequencies based on positive cultures can be extrapolated to the resistance frequency in invasive aspergillosis.

Epidemiological studies regarding other aspergillus diseases are lacking in the Netherlands. Estimates of the rate of CPA were based on the number of patients with tuberculosis and the assumption that 25% of the cases of CPA are related to tuberculosis. However, the percentage of CPA cases that are actually related to tuberculosis is highly dependent on the rate of tuberculosis in the country. The Netherlands has a relatively low rate of tuberculosis (5 per 100 000), and a high rate of COPD which is associated with about 35% of CPA cases in other series and other relevant underlying conditions like sarcoidosis were not taken into account; hence, our calculations may underestimate the actual burden of CPA.^43^, ^44^ The estimated annual incidence of *Fusarium* keratitis is 8. However, this estimate is based on the mean incidence between 2005 and 2016. Interestingly, the incidence of *Fusarium* keratitis has been increasing over the years and 15‐25 annual cases were found in 2014 to 2016.^19^ Similar trends are seen in Germany.^45^ Thus, the estimated incidence of 8 may underestimate the current incidence of *Fusarium* keratitis.

As of January 2018, 20 800 patients were confirmed HIV positive of whom 93% (19 289 patients) were on antiretroviral therapy. Over the years, an increasing percentage of patients received antiretroviral therapy in the Netherlands^3^ and since 2008 the number of new HIV diagnoses has declined each year (n = 724 for 2017).^3^ Currently, an estimated 2300 cases are considered undiagnosed HIV positive individuals in the Netherlands. This decline in HIV patients resulted in a relative decline of PJP and extrapulmonary cryptococcosis in HIV patients.

As described by van Elden et al (2000),^46^ the national incidence of cryptococcosis in non‐HIV patients between the year 1986 and 2000 ranged between one and six cases annually, with an average of 5.33 between 1997 and 1999. More recently, the Netherlands Reference Laboratory for Bacterial Meningitis (AMC/RIVM)^31^, ^32^ published data from the years 2015‐2017, demonstrating an average incidence of 9.33 (range 9‐10) patients with HIV and non‐HIV cases combined. This increase could be partially attributable to the ever increasing number of solid organ transplants in the Netherlands, totalling 202 in the year 2000^47^ and 1270 in 2017.^8^

*Pneumocystis jirovecii* pneumonia patients in the Netherlands have been primarily treated with corticosteroids as their main risk factor. The immunosuppressive properties of corticosteroids, biologicals and chemotherapy are a known risk factor for PJP.^48^, ^49^ Studies have described PJP incidences in solid organ transplantation patients in Switzerland (1.4%)^50^ and global incidences (including the Netherlands) in recipients of first allogeneic and autologous HSCTs (0.63% and 0.28%, respectively).^51^ However, published data on PJP diagnosed patients using corticosteroid and other immunosuppressive drugs in the Netherlands are to our knowledge unavailable. Of note, PJP prophylaxis is prescribed for acute lymphocytic leukaemia, allogeneic HSCT, treatment with alemtuzumab, fludarabine/cyclophosphamide/rituximab combinations, >4 weeks of treatment with corticosteroids and well‐defined primary immune deficiencies in children.^52^ Combining PJP estimates for the HIV, HSCT and solid organ transplant cohorts alone would underestimate the total PJP burden. Therefore, we retrospectively evaluated local PJP incidences and extrapolated these nationwide, thereby finding that only 6.5% of PJP cases are HIV‐associated.

Candidemia and *Candida* peritonitis numbers are based on European averages for ICU admissions in (almost) exclusively University Medical Centres.^26^ To our knowledge, comprehensive data from the Netherlands alone is currently unavailable. Candidemia incidences outside the ICU are not accounted for, although incidences are possibly over 10 times lower^53^ and might overlap with subsequent ICU admissions. In addition*, Candida* oesophagitis, mucocutaneous candidiasis and other less common infections are not included in our estimate. Thus, the number of serious infections due to *Candida* listed here may underestimate the national burden.

The incidence of vulvo‐vaginal candidiasis in several European countries and USA combined was estimated to be 9% in 2013^28^ in women aged 16 to 65 years. Overall rates published were based on self‐reporting to a healthcare provider‐diagnosed vaginal yeast infection, and survey completion rates were very low. Besides the self‐reporting and probable selection bias, general practitioners have been reported to over‐diagnose vulvo‐vaginal candidiasis.^29^ Thus, we believe 9% is an overestimation of the actual incidence of vulvovaginal candidiasis and therefore we used 6% in our calculations.

Estimates of endemic mycoses could not be made as surveillance data was not available and the rate is highly correlated to travel and migration to and from endemic regions for which no accurate estimates could be made. Furthermore, data on mucocutaneous candidiasis, non‐*Fusarium* fungal keratitis, rare invasive yeast infections and non‐*Aspergillus* invasive fungal infections are lacking and estimates could not be made.

The total burden of fungal diseases in the Netherlands was estimated to be 254 491 patients yearly, encompassing approximately 1.5% of the country's population. This includes patients with invasive aspergillosis, invasive candidiasis, PJP, cryptococcosis, and mucormycosis. Of note, 145 patients (11.3%) with invasive aspergillosis are estimated to be infected with an azole resistant isolate. Some azole resistance is likely in patients with chronic and allergic aspergillosis and could affect 29 patients with CPA, a figure that needs validation with data. Furthermore, the less severe syndromes ABPA and SAFS, and recurrent *Candida* vaginitis were also included due to their significant impact on the quality of life. The total number of serious fungal infections was 3,185 annually. However, the number of serious infections listed here may be an underestimate of the national burden and are not to be considered all‐encompassing. Some serious infections like phaeohyphomycosis, endemic mycoses and dermatophytosis could not be included due to the unavailability of surveillance data. Most of the estimates were based on extrapolations due to the unavailability of surveillance data. With emerging new risk groups and increasing reports on antifungal resistance, surveillance programmes are warranted to obtain more accurate estimates of fungal disease epidemiology and associated morbidity and mortality.

## CONFLICTS OF INTEREST

The authors declare no conflict of interest.

## AUTHOR CONTRIBUTIONS

DD and JM involved in conceptualisation; JB, EM and DD involved in methodology; JB and EM involved in validation; JB and EM involved in formal analysis; JB and EM wrote the original draft preparation; DD, PV and JM wrote, reviewed and edited the manuscript; PV and JM involved in supervision. All authors have read and agreed to the published version of the manuscript.
